# Multigenerational effects of fetal-neonatal iron deficiency on hippocampal BDNF signaling

**DOI:** 10.1002/phy2.96

**Published:** 2013-10-02

**Authors:** Mariah B Blegen, Bruce C Kennedy, Katie A Thibert, Jonathan C Gewirtz, Phu V Tran, Michael K Georgieff

**Affiliations:** 1 Department of Pediatrics, University of MinnesotaMinneapolis, 55455, Minnesota; 2 Graduate Program in Neuroscience, University of MinnesotaMinneapolis, 55455, Minnesota; 3 Department of Psychology, University of MinnesotaMinneapolis, 55455, Minnesota

**Keywords:** Development, epigenetics, JARID, nutrition, rat

## Abstract

Fetal-neonatal iron deficiency induces adult learning impairments concomitant with changes in expression of key genes underlying hippocampal learning and memory in spite of neonatal iron replenishment. Notably, expression of brain-derived neurotrophic factor (BDNF), a gene critical for neuronal maturation and synaptic plasticity, is lowered both acutely and in adulthood following early-life iron deficiency. Although the mechanism behind its long-term downregulation remains unclear, epigenetic modification in BDNF, as seen in other models of early-life adversity, may play a role. Given that early iron deficiency occurs during critical periods in both hippocampal and gonadal development, we hypothesized that the iron-sufficient offspring (F2 IS) of formerly iron-deficient (F1 FID) rats would show a similar suppression of the BDNF gene as their parents. We compared hippocampal mRNA levels of BDNF and functionally related genes among F1 IS, F1 ID, and F2 IS male rats at postnatal day (P) 15 and P65 using RT-qPCR. As expected, the F1 ID group showed a downregulation of BDNF and associated genes acutely at P15 and chronically at P65. However, the F2 IS group showed an upregulation of these genes at P15, returning to control levels at P65. These results demonstrate that adverse effects of early iron deficiency on hippocampal gene expression observed in the F1 are not present in the F2 generation, suggesting differential effects of nutritionally induced epigenetic programing during the critical periods of hippocampal and gonadal development.

## Introduction

Iron deficiency is the most prevalent micronutrient deficiency in the world, affecting at least 2 billion people worldwide (World Health Organization (WHO) 1998). It has been estimated that ∼20–30% of pregnancies are iron deficient (ID), with up to 80% prevalence in low- and middle-income countries (Black et al. 2011). Iron deficiency during the fetal-neonatal period can cause acute and long-lasting learning and memory impairments in humans in spite of prompt early neonatal iron replenishment (Lozoff et al. 2006). These deficits have been phenocopied in the rodent model of early iron deficiency (Felt et al. 2006; Schmidt et al. 2007; Carlson et al. 2009), where the deficits have been ascribed to abnormalities in myelination, monoaminergic and glutamatergic neurotransmission, hippocampal morphology and metabolism, and gene expression (Connor and Menzies 1996; Beard et al. 2003a,b; Rao et al. 2003; Jorgenson et al. 2005; Carlson et al. 2007; Tran et al. 2008; Brunette et al. 2010). All of these dysfunctions have been shown to persist beyond the period of early-life iron deficiency (Tran et al. 2009; Rao et al. 2011; Unger et al. 2012).

Previously we demonstrated that fetal-neonatal iron deficiency dysregulated genes important for hippocampal development both acutely and persistently into early adulthood (Tran et al. 2009). One of these key genes is brain-derived neurotrophic factor (BDNF). BDNF is a nerve growth factor that signals through its cognate receptor trkB to mediate neuronal maturation (e.g., dendritic growth and the formation of synapses), synaptic plasticity (Dragunow et al. 1997; Shimada et al. 1998; Huang and Reichardt 2001; Hennigan et al. 2007), and processes important for hippocampal-dependent learning and memory (Hall et al. 2000; Xu et al. 2000; Heldt et al. 2007; Yoshii and Constantine-Paton 2007). Thus, reduced BDNF signaling seen in the formerly iron-deficient (FID) adult rat could underlie the persistent cognitive impairments. The mechanisms underlying the downregulation of BDNF remain unclear, but one possibility of long-term dysregulation is that of stable epigenetic modifications (Branchi et al. 2004).

Epigenetics refers to the process of gene regulation independent of changes in the genetic code, including DNA methylation and posttranslational modifications of histone complexes that activate or silence gene expression (Kouzarides 2007; Vernimmen et al. 2011). Epigenetic modifications of the genome provide mechanisms that allow the stable propagation of gene activity states from one generation of cells to the next (Youngson and Whitelaw 2008; Roth et al. 2011). The propagation of epigenetic modifications is one proposed mechanism for the transgenerational changes in gene expression and behavior observed in models of early-life adversity. For example, rats subjected to early maltreatment showed more maltreatment behaviors to their own offspring (Roth et al. 2009) and the transmission of this behavior is accompanied by stable, epigenetic changes to the BDNF gene in the hippocampus and other brain areas (Roth et al. 2010).

Fetal-neonatal iron deficiency persistently suppresses BDNF expression in the adult rat hippocampus (Tran et al. 2009), suggesting a possible role for epigenetic modifications. Furthermore, germ cell progenitors in the developing fetus are similarly exposed to an iron-deficient (ID) environment, allowing for the possibility of heritable epigenetic modifications to the germ cell line. To address these possibilities we sought to determine if BDNF expression and signaling would also be suppressed in the iron-sufficient offspring (F2 IS) of FID rats. We also evaluated the expression of Jumonji domain –ARID-containing protein (JARID), a family of histone demethylases, in which functionality is confirmed by an iron-binding jumonji domain (Takeuchi et al. 2006). JARIDs may be a key upstream regulator of BDNF expression, thus providing a direct link between iron status and BDNF gene regulation.

## Material and Methods

### Animals

**Figure 1:**
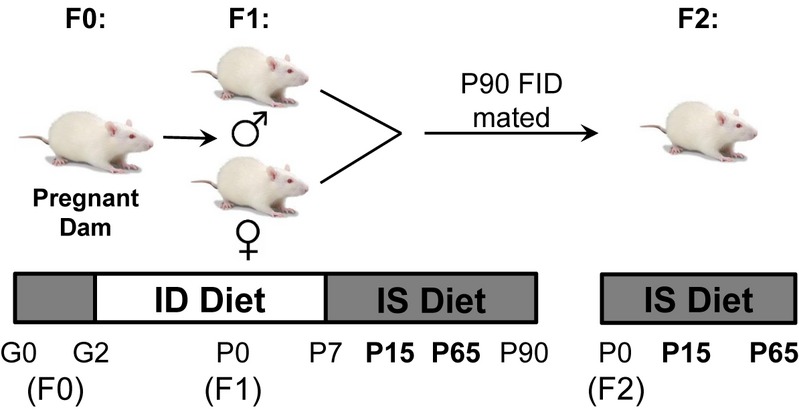
Mating and diet scheme required to produce generation of F2 iron-sufficient (F2 IS) from formerly iron-deficient (FID) rats. Pregnant dams (P0) were given iron-deficient (ID) diet beginning at gestational day (G) 2. Nursing dams and F1 pups continued on an ID diet until postnatal day (P) 7, at which time both were switched to an IS diet. F1 rats were weaned at P21. At P90, when no longer iron-deficient (formerly iron-deficient) F1 were allowed to mate to produce F2 pups. Hippocampal RNA was collected and evaluated from F1 and F2 rats at the P15 and P65 time points shown in bold.

Sprague Dawley® rats (Charles River, Wilmington, MA) were kept on a 12 h light:dark cycle at room temperature. Control and experimental animals were generated as illustrated in Figure 1. In brief, to generate the F1 fetal-neonatal ID group, pregnant rat dams were given a purified ID diet (4 mg/kg iron, Harlan-Teklad, Madison, WI) from gestational day (G) 2 through postnatal day (P) 7. At P7, nursing dams were switched to a purified IS diet (200 mg/kg iron, Harlan-Teklad), and at this point, the ID pups constituted the F1 ID group. By P56, the F1 ID offspring were no longer ID and had normal brain iron concentrations (Rao et al. 2010). They are therefore labeled as the F1 FID group. At P90, the F1 FID rats were mated (non littermate males and females) to generate F2 offspring, who never received ID diet (F2 IS). The true control group was generated from IS control dams, who were given the IS diet throughout the experiment, giving rise to F1 IS pups. Thus, the three comparison groups were F1 IS, F1 ID, and iron-sufficient F2 offspring (F2 IS) of P90 F1 FID. All litters were culled to eight pups (four to six males complemented with females) at birth to ensure uniformity across litters and were weaned at P21. Animals had free access to food and water. All experiments were approved by the University of Minnesota Institutional Animal Care and Use Committee.

### Hippocampus collection

P15 and P65 male rats were sacrificed and the brains were immediately removed and placed on filter paper with cold phosphate-buffered saline (PBS) atop a metal heat-block chilled on ice. Brains were bisected along the midline and the hippocampi were dissected. The tissue was flash frozen in liquid nitrogen and stored at −80°C.

### RNA isolation and cDNA synthesis

RNA was extracted from hippocampal tissue taken from the right hemisphere using RNAqueous® −4PCR total RNA isolation kit (Ambion®, Austin, TX). Each hippocampus was lysed in 800 μL Lysis Binding Solution. A quantity of 400 μL RNA lysate was further processed, while the other 400 μL was stored at −80°C. RNA was isolated from lysate following the manufacturer's instructions. RNA concentration and quality were analyzed using a NanoDrop-2000 spectrophotometer. 0.8–1.0 μg of total RNA was used to generate cDNA using a High Capacity RNA-to-cDNA™ kit by Applied Biosystems (Carlsbad, CA). cDNA was then diluted 10-fold with diethylpyrocarbonate (DEPC)-treated water and stored at −20°C.

### Real-time quantitative PCR

The real-time quantitative PCR (RT-qPCR) experiments were run with both singleplex probes and multiplex probes optimized for the rat. For the singleplex experiments, each reaction contained half the manufacturer-recommended volume: 4 μL diluted cDNA, 5 μL 2X Taqman® qPCR Universal Mix, 0.5 μL 20X Taqman® Gene Expression Assay, and 0.5 μL DEPC-treated water (Applied Biosystems, Carlsbad, CA) in a 96-well plate. The control probe/primer used was 45S preribosomal RNA (Assay ID Rn03928990_g1) and the probe/primer for gene of interest was in separate wells. Selection of gene analyzed is listed in Table 1. For duplex RT-qPCR, method validation was performed by comparing data from uniplex and duplex experiment. The duplex experiment was used to minimize replication error and was optimized in 20 μL reactions following validation test. Thermocycling was completed according to manufacturer's protocol (Applied Biosystems) using a MX3000P instrument (Stratagene, La Jolla, CA).

**Table 1:** List of Real-Time PCR Taqman™ Probes

Gene name	Gene bank accession #	ABI assay ID	Category
*BDNF-III*	BC087634	Rn01484928_m1	Signaling molecule
*BDNF-IV*	NM_012513	Rn02531967_s1	Signaling molecule
*Camk2α*	NM_012920.1	Rn01258147_m1	Kinase
*Cfl1*	NM_017147.2	Rn00820797_g1	Cytoplasmic protein
*Cxcl12*	NM_001033882.1	Rn0573260_m1	Signaling molecule
*Dlgh4*	NM_019621.1	Rn00571479_m1	Postsynaptic density protein
*Dusp4*	NM_022199	Rn00573501_m1	Phosphatase
*Egr1*	NM_012551.2	Rn00561138_m1	Transcription factor
*Egr2*	NM_053633.1	Rn00586224_m1	Transcription factor
*Gda*	NM_031776.2	Rn00582297_m1	Enzyme
*Gria1*	NM_031608.1	Rn00709588_m1	Neurotransmitter receptor
*Gria2*	NM_001083811.1	Rn01451959_m1	Neurotransmitter receptor
*Grin1*	NM_017010.1	Rn01436038_m1	Glutamate receptor
*Grin2a*	NM_012573.3	Rn00561341_m1	Glutamate receptor
*Grin2b*	NM_012574.1	Rn00680474_m1	Glutamate receptor
*Hif1α*	NM_024359.1	Rn00577560_m1	Transcription factor
*Hmgcr*	NM_013134.2	Rn00565598_m1	CoA reductase
*Igf1*	NM_001082477.2	Rn00710306_m1	Signaling molecule
*Igf2*	NM_001190162.1	Rn01454518_m1	Signaling molecule
*JHDM1D*	NM_001108253.1	Rn01754458_m1	Histone demethylase
*Jarid1b* (*Kdm5b*)	NM_006618.3	Rn01758879_m1	Histone demethylase
*JMJD3 (Kdm6b*)	NM_001108829.1	Rn0471506_m1	Histone demethylase
*Nr3c1* (*GR*)	NM_012576.2	Rn00561369_m1	Hormone receptor
*Ntrk2* (*trkB*)	NM_012731	Rn01441747_m1	Membrane receptor
*Pfn1*	NM_022511.2	Rn04219475_g1	Structural protein
*Rn45s*	NR_046239.1	Rn3928990_g1	Internal control
*Vegfα*	NM_001110333.1	Rn0011160_m1	Signaling molecule

### Statistics

Graphs and statistical analyses were generated using Prism GraphPad (GraphPad Software Inc., San Diego, CA). Data were analyzed using unpaired *t*-tests and one-way analysis of variance (ANOVA) with significance set at *P* < 0.05.

## Results

### Increased hippocampal brain-derived neurotrophic factor signaling in P15 F2 males

**Figure 2:**
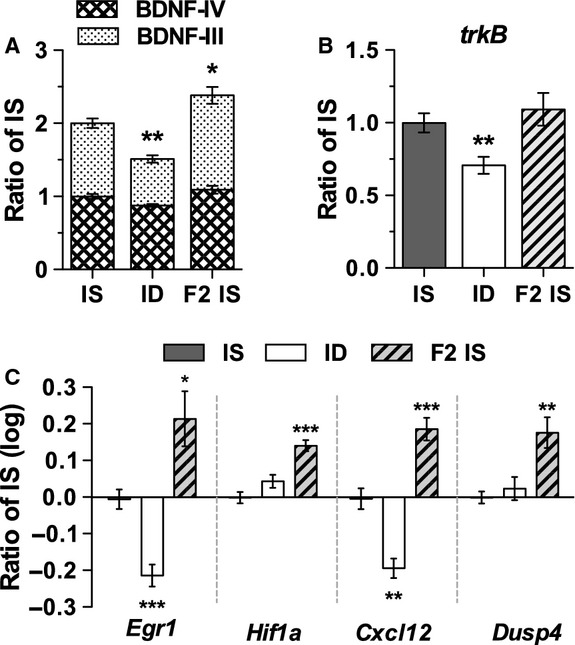
Quantitative PCR (qPCR) analysis demonstrates increased expression of BDNF and downstream targets in the P15 F2 IS rats. Transcript levels were measured from the hippocampus of F1 iron sufficient (IS), F1 iron deficient (ID), and F2 iron-sufficient (F2 IS) rats for (A) BDNF-III and BDNF-IV, (B) trkB, and (C) Egr1, Hif1a, Cxcl12, and Dusp4. Values are means ± SEM; *n* = 4–6/group; *P < 0.05, **P < 0.01, and ***P < 0.001, Unpaired *t*-test.

Confirming our previous report (Tran et al. 2008), BDNF expression in the P15 ID hippocampus was downregulated, without a compensatory increase in its receptor, trkB (Fig. 2, ID). In contrast, in the F2 offspring of the FID rats BDNF expression was upregulated, while expression of trkB did not differ significantly from F1 IS control (Fig. 2, F2 IS). BDNF signaling mediates a variety of activity-dependent immediate early genes and transcription factors that contribute to hippocampal development and function (Alder et al. 2003; Calella et al. 2007). To assess the effect of increased BDNF and trkB expression, we analyzed the expression of genes downstream of BDNF signaling. P15 F2 IS hippocampus showed elevated expression of downstream effectors of BDNF signaling such as Egr1, Hif1a, Cxcl12 and Dusp4 (Fig. 2C), confirming the increased BDNF activity.

### Increased expression of genes augmenting neuronal differentiation in the P15 F2 IS developing hippocampus

To provide further evidence of the functional effect of increased BDNF signaling in the F2 IS offspring of FID rats, we quantified mRNA levels of genes that facilitate neuronal growth and maturation in the P15 developing hippocampus. Compared to the F1 IS control group, F2 IS group showed upregulation of genes implicated in processes important for synaptic plasticity, including cellular growth (*Egr1, Egr2*), dendritogenesis and synaptogenesis (*Cfl1, Pfn1, Gda, Dlgh4, Vegfa, CamKIIa, Gria*, and *Grin*), and cholesterol synthesis (*Hmgcr*) (Table 2). Most of these same genes were downregulated in the P15 F1 ID group (Table 2), which is consistent with previous findings (Tran et al. 2009) and demonstrates differential effects across the two generations.

**Table 2:** Real-Time PCR Analysis of P15 Male Rat Hippocampus

Transcript	IS	ID	F2 IS
*CamkIIa*	1.00 ± 0.26 (5)	0.63 ± 0.12* (6)	1.38 ± 0.28 (5)
*Cfn1*	1.00 ± 0.19 (4)	0.58 ± 0.08** (5)	1.65 ± 0.27** (5)
*Dlgh4*	1.00 ± 0.21 (6)	0.61 ± 0.09** (5)	1.69 ± 0.39** (5)
*Egr2*	1.00 ± 0.25 (5)	0.51 ± 0.10** (5)	1.5 ± 0.28* (4)
*Gda*	1.00 ± 0.31 (6)	0.54 ± 0.17** (6)	1.75 ± 0.30** (5)
*GR*	1.00 ± 0.12 (5)	1.56 ± 0.41** (4)	1.05 ± 0.14 (5)
*Gria1*	1.00 ± 0.14 (5)	1.00 ± 0.16 (6)	1.26 ± 0.15* (5)
*Gria2*	1.00 ± 0.13 (5)	1.04 ± 0.14 (6)	1.23 ± 0.14* (5)
*Grin1*	1.00 ± 0.24 (6)	0.62 ± 0.06** (5)	1.22 ± 0.26 (4)
*Grin2a*	1.00 ± 0.13 (5)	0.67 ± 0.11** (5)	2.00 ± 0.54** (4)
*Grin2b*	1.00 ± 0.16 (5)	0.54 ± 0.12*** (6)	1.82 ± 0.32** (4)
*Igf1*	1.00 ± 0.08 (5)	1.06 ± 0.13 (6)	1.10 ± 0.13 (5)
*Hmgcr*	1.00 ± 0.20 (5)	1.12 ± 0.11 (5)	1.88 ± 0.23*** (5)
*Pfn1*	1.00 ± 0.14 (6)	0.64 ± 0.04*** (5)	1.60 ± 0.37** (5)
*Vegfα*	1.00 ± 0.24 (5)	1.03 ± 0.25 (6)	1.56 ± 0.36* (5)

Values are mean ± SD. Sample sizes are in parenthesis.

* *P* < 0.05,

** *P* < 0.01,

*** *P* < 0.001, Unpaired *t*-test.

### Normalization of brain-derived neurotrophic factor signaling in the young adult P65 F2 IS hippocampus

To determine whether the increased BDNF signaling persists into early adulthood of the F2 generation, we analyzed expression of BDNF and its downstream signaling targets at P65. As before (Tran et al. 2009), BDNF expression and signaling continued to be suppressed in the F1 FID hippocampus (Table 3) compared to the F1 IS control. In contrast, F2 IS animals showed normalization of BDNF signaling in adulthood (Table 3), suggesting that the increase in BDNF in this group occurs only during hippocampal differentiation.

**Table 3:** Real-Time PCR Analysis of P65 Male Rat Hippocampus

Transcript	IS	FID	F2 IS
*BDNF-III*	1.00 ± 0.10 (5)	0.72 ± 0.15** (7)	0.97 ± 0.07 (5)
*Camk2α*	1.00 ± 0.36 (8)	1.16 ± 0.22 (9)	1.18 ± 0.36 (5)
*Cfl1*	1.00 ± 0.14 (4)	0.52 ± 0.23** (7)	0.89 ± 0.31 (5)
*Cxcl12*	1.00 ± 0.14 (6)	0.33 ± 0.13*** (5)	0.99 ± 0.20 (5)
*Dusp4*	1.00 ± 0.19 (6)	0.59 ± 0.29* (7)	0.68 ± 0.19* (5)
*Egr1*	1.00 ± 0.19 (6)	0.93 ± 0.26 (4)	0.65 ± 0.04** (5)
*GR*	1.00 ± 0.26 (6)	0.95 ± 0.0.4 (5)	1.32 ± 0.26 (5)
*Grin2b*	1.00 ± 0.35 (8)	0.61 ± 0.23* (10)	1.19 ± 0.41 (5)
*Hmgcr*	1.00 ± 0.05 (6)	0.41 ± 0.05*** (5)	0.90 ± 0.06 (5)
*Igf1*	1.00 ± 0.35 (8)	1.148 ± 0.50 (10)	1.15 ± 0.37 (5)
*Igf2*	1.00 ± 0.71 (8)	0.58 ± 0.19 (9)	0.69 ± 0.24 (5)
*Pfn1*	1.00 ± 0.20 (6)	0.66 ± 0.24* (6)	0.92 ± 0.12 (5)
*trkB*	1.00 ± 0.10 (6)	0.46 ± 0.14*** (5)	0.77 ± 0.09 **(5)

Values are mean ± SD. Sample sizes are in parentheses.

* *P* < 0.05,

** *P* < 0.01,

*** *P* < 0.001, Unpaired *t*-test.

### Upregulation of iron-containing histone H3 demethylases in P15 F2 IS

**Figure 3:**
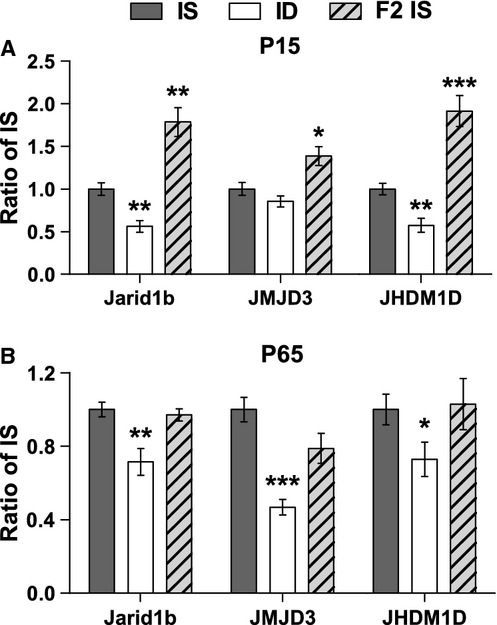
Transient upregulation of Jumonj-domain containing histone demethylases (JARIDs) in the F2 IS rats. qPCR was used to quantify hippocampal transcript levels of Jarid1b (kdm5b), JMJD3 (kdm6b), and JHDM1D at P15 (A) and P65 (B). Values are means ± SEM; *n* = 4–7; *P < 0.05, **P < 0.01, and ***P < 0.001, Unpaired *t*-test.

To provide a potential link between fetal-neonatal iron status and iron-dependent chromatin modifiers and to gain insight into a possible mechanism underlying BDNF gene regulation during iron deficiency, we analyzed expression of histone H3 demethylases (JARIDs) that require iron as a cofactor (Takeuchi et al. 2006; Sengoku and Yokoyama 2011). All analyzed JARIDs were reduced in the F1 ID hippocampus at P15 and in the F1 FID hippocampus P65 (Fig. 3, ID/FID), suggesting a potential role in BDNF suppression. In contrast, the F2 IS group showed an acute upregulation of *Jarid1b (Kdm5b), JMJD3 (Kdm6b)*, and *JHDM1d* in the developing P15 hippocampus (Fig. 3A), and, like BDNF, all three JARIDs were restored to normal levels by P65 (Fig. 3B).

## Discussion

Fetal-neonatal iron deficiency is a global health concern because it results in long-lasting abnormalities in cognitive function and affective behaviors (Schmidt et al. 2004; Siddappa et al. 2004; Lozoff et al. 2006; Li et al. 2009; Christian et al. 2010; Chang et al. 2013). These effects have been replicated in animal models, enabling further investigation into the molecular bases underlying the persistent defects that include monoamine signaling, myelination, neural metabolism, and gene expression (Connor and Menzies 1996; Beard et al. 2003b; Tran et al. 2009; Rao et al. 2011). These long-term findings imply that iron has an important role in the programing of genes that are critical for normal brain development and function. Reprogramming of gene expression by early-life iron deficiency could involve stable epigenetic modifications that impact multiple generations. From an epidemiological perspective, this would be highly disadvantageous for populations where iron deficiency is endemic and where significant attempts are being made to improve their iron status.

One possible mechanism for the transmission of phenotypes across multiple generations, which has been observed in models of adverse prenatal environments (Roth et al. 2009, 2010), is stable epigenetic changes in the F1 developing germ cells. Primordial germ cell development occurs in utero and is sensitive to environmental perturbations (Skinner and Anway 2005). Thus, early iron deficiency could impact their epigenetic programing. This study addressed whether early iron deficiency alters BDNF expression across generations. On the basis of the existing literature (Roth et al. 2009; Tran et al. 2009; McGowan et al. 2011), we hypothesized that early-life iron deficiency persistently alters hippocampal BDNF expression in the F2 offspring of FID rats. Contrary to our hypothesis, the findings revealed the opposite effect, specifically a gain-of-BDNF function accompanied by the upregulation of genes implicated in neuronal growth and differentiation in the F2 generation. These effects were transient but occurred at an important time in terms of hippocampal differentiation. Collectively, the findings reveal that early iron deficiency does not result in a lasting dysregulation of BDNF across multiple generations and suggest that early iron deficiency does not lead to heritable epigenetic modifications of BDNF regulation and function. In spite of the transient nature of increased BDNF expression in the F2 generation, the finding may be functionally important because it occurred during a critical period of hippocampal differentiation (Fretham et al. 2011; Callahan et al. in press). Increased BDNF activity can be potentially positive for the developing hippocampus through its role of facilitating neuronal dendritogenesis and synaptogenesis via upregulation of genes promoting neuronal dendrite growth and branching (Table 2). Increased BDNF and its downstream effects on synaptogenesis are associated with beneficial behavioral outcomes, even if the increase in BDNF expression is only transient. For example, offspring of high licking and grooming dams show increased BDNF expression at P8 relative to low licking and grooming dams (Liu et al. 2000). Although BDNF expression had normalized by P18, offspring of high licking and grooming dams exhibited better spatial memory on the Morris water maze as adults (Liu et al. 2000). Future studies could explore whether the early surge in BDNF observed in F2 IS pups produces an acute or prolonged effect on learning and memory in F2 pups. Alternatively, higher levels of BDNF during hippocampal maturation could also lead to a more rapid closure of the critical period as has been shown in the visual cortex (Huang et al. 1999). Taken together, greater plasticity resulting from increased synaptogenesis could be offset by the decrease in plasticity associated with an early closure of the critical period. Whether these molecular changes in the F2 pups would translate to beneficial effects in terms of hippocampal function remain to be determined.

Increased BDNF signaling during the peak of neuronal differentiation in the hippocampus (Pokorny and Yamamoto 1981; Avishai-Eliner et al. 2002) of the F2 generation was unexpected given the findings of lower BDNF expression in the F2 generation of other models of early-life adversity. Given its occurrence during the nursing phase, we speculate that maternal care could be a contributing factor. Maternal-infant interactions can influence BDNF regulation, although the nature of this relationship remains controversial with conflicting reports suggesting either a positive effect (Liu et al. 2000; van Hasselt et al. 2012; Branchi et al. 2013), no effect (Blaze et al. 2013; Kosten et al. 2013), or a negative effect (Dalle Molle et al. 2012). In a preliminary cross-fostering experiment, F2 pups raised by IS control dams did not exhibit the upregulation of BDNF-III observed in uncross-fostered F2 pups at P15 (B. C. Kennedy, unpubl. obs.). Importantly, this suggests that maternal-infant interactions could contribute to early differences in gene expression between F2 IS and ID pups.

Consistent with our previous findings, expression of BDNF and many downstream targets was downregulated in P65 FID animals relative to IS controls (Tran et al. 2009). However, certain genes exhibited a deviation from this trend and are intriguing in terms of complex gene-environment interactions. For example, Egr1 and Cxcl12 showed a different regulatory response compared to Hif1a and Dusp4 in P15 F1 ID and F2 IS. The small upregulation of Hif1a during iron deficiency anemia (P15 ID) was likely driven by cellular hypoxia, whereas its upregulation in the F2 IS could be driven by BDNF/TrkB signaling (Nakamura et al. 2006). Likewise, the upregulation of Dusp4, a negative regulator of MAPK activity, in the F2 IS was likely mediated by changes in Egr1 expression (Berasi et al. 2006). Furthermore, the finding that a number of genes, including TrkB and Dusp4, reverted to the levels of F1 FID at P65 highlights the transient nature of increased BDNF signaling in the F2 hippocampus. It remains to be seen whether other genes show similarly transient transgenerational epigenetic programing effects.

Finally, expression of JARIDs at both P15 and P65 time points parallels the changes seen in BDNF in the F2 IS and ID treatment groups. Although JARID expression may be modulated by iron availability, it is less clear how early iron deficiency affects long-term expression of JARIDs in the FID hippocampus. The continued downregulation of JARIDs after iron repletion in P65 FID rat suggests that iron deficiency is unlikely to alter JARID expression directly, but rather via a secondary mechanism such as epigenetic modifications of the JARIDs themselves. The fact that JARIDs and BDNF gene were both upregulated at P15 in F2 animals, which were IS, further supports the notion that alterations in regulation occur via epigenetic mechanisms. Considering that iron is a key regulator of JARIDs' activity, the relationship between BDNF expression and JARIDs could imply that JARIDs modulate transcriptional activity of BDNF and related genes through demethylation at specific promoter regions. Early-life iron deficiency nonspecifically suppressed expression of different types of JARIDs that exhibit opposite effects on overall levels of transcription. For instance, lower JARID1b (Kdm5b), which removes the methyl group from histone H3 lysine 4 trimethyl (H3K4me3), could lead to a less transcriptionally active chromatin. Conversely, lower JMJD3 (Kdm6b) and JHDM1d, which respectively, remove methyl groups from H3K9me3 and H3K27me3, could result in a less transcriptionally repressive conformation (Kouzarides 2007; Pasini et al. 2010; Vernimmen et al. 2011). Thus, lower expression of both JARID subtypes in the ID and FID hippocampus likely results in a complex balance between activating and repressing chromatin structures. The fact that we found more genes with increased transcriptional activity (Table 2) in this study suggests overall more “active” chromatin conformations in the P15 F2 hippocampus. Systemic analysis of chromatin structure (ChIP-seq) of early ID hippocampus would provide important insights into the epigenetic programing effects of early ID and its impact across generations.

### Significance and perspectives

The concept of the developmental origins of adult health and disease has gained substantial support in recent years. For the most part, this concept addresses how early-life events affect health outcomes in adulthood within the same generation. Of more recent concern is the transmission of phenotypes and associated epigenetic modifications across multiple generations demonstrated in various models of early-life adverse environment, including nutritional deficiencies. Given the prevalence of fetal-neonatal iron deficiency worldwide, it is encouraging that the long-term negative impacts on the expression of genes relevant to cognitive function do not appear to affect the next generation. The lack of multigenerational effects in our model stands in contrast with other models of early-life adversity (e.g., maternal stress, infant abuse and neglect, maternal dietary restrictions), suggesting distinct etiologies and underlying regulatory mechanisms (e.g., transient vs. stable or reversible vs. irreversible epigenetic modifications). Thus, further investigation into the epigenetic and molecular basis of long-term effects of gene suppression in the FID rats may provide important insights into adjuvant therapeutic strategies to complement iron therapy including supplemental diets enriched in methyl donors (e.g., choline, betaine).
